# Enteroviral Pathogenesis of Type 1 Diabetes: The Role of Natural Killer Cells

**DOI:** 10.3390/microorganisms8070989

**Published:** 2020-07-01

**Authors:** Magloire Pandoua Nekoua, Arthur Dechaumes, Famara Sane, Enagnon Kazali Alidjinou, Kabirou Moutairou, Akadiri Yessoufou, Didier Hober

**Affiliations:** 1 Laboratoire de Virologie ULR3610, Univ Lille, CHU Lille, F-59000 Lille, France; pamanek88@gmail.com (M.P.N.); a.dechaumes@gmail.com (A.D.); famara.sane@chru-lille.fr (F.S.); enagnonkazali.alidjinou@chru-lille.fr (E.K.A.); 2 Laboratoire de Biologie et Physiologie Cellulaires, Institut des Sciences Biomédicales Appliquées (ISBA), Faculté des Sciences et Techniques (FAST), Université d’Abomey-Calavi, Cotonou 01 BP 526, Benin; kabirou.moutairou@gmail.com (K.M.); akadiri.yessoufou@gmail.com (A.Y.)

**Keywords:** enteroviruses, NK cells, HLA class I, persistence, type 1 diabetes

## Abstract

Enteroviruses, especially group B coxsackieviruses (CV-B), have been associated with the development of chronic diseases such as type 1 diabetes (T1D). The pathological mechanisms that trigger virus-induced autoimmunity against islet antigens in T1D are not fully elucidated. Animal and human studies suggest that NK cells response to CV-B infection play a crucial role in the enteroviral pathogenesis of T1D. Indeed, CV-B-infected cells can escape from cytotoxic T cells recognition and destruction by inhibition of cell surface expression of HLA class I antigen through non-structural viral proteins, but they can nevertheless be killed by NK cells. Cytolytic activity of NK cells towards pancreatic beta cells persistently-infected with CV-B has been reported and defective viral clearance by NK cells of patients with T1D has been suggested as a mechanism leading to persistence of CV-B and triggering autoimmunity reported in these patients. The knowledge about host antiviral defense against CV-B infection is not only crucial to understand the susceptibility to virus-induced T1D but could also contribute to the design of new preventive or therapeutic approaches for individuals at risk for T1D or newly diagnosed patients.

## 1. Introduction

Type 1 diabetes (T1D) is a chronic autoimmune disease that results from the selective destruction and loss of functional insulin-producing pancreatic islet beta cells occurring in genetically predisposed individuals and probably triggered or accelerated by environmental factors such as drugs, toxins, nutrients and viruses [1,2,3,4]. Several epidemiological and clinical data support the hypothesis that enteroviral infections are linked with the development of islet autoimmunity or onset and progression of clinical T1D [4,5,6,7,8].

Enteroviruses (EVs) are small (from 25 to 30 nm in diameter), non-enveloped with an icosahedral capsid, positive-sense single-stranded RNA genome viruses belonging to the *Picornaviridae* family [9]. The genus *Enterovirus* is very important in medicine and encompasses seven species involved in human diseases (*Human Enterovirus* A–D and *Human Rhinovirus* A–C) and over 250 serologically distinct viruses [9,10]. These ubiquitous pathogens across the world are transmitted mainly by the fecal–oral and respiratory routes and can infect a wide range of tissues [11,12]. Even though most enteroviral infections remain asymptomatic, they have been associated with a wide spectrum of clinical signs ranging from relatively mild symptoms such as fever, gastro-enteritis, skin lesions and headache to severe acute forms such as meningitis, hepatitis, encephalitis, myocarditis, pancreatitis and hand, foot and mouth disease [10,12,13,14,15]. In addition to these severe acute clinical features, enteroviral infections, especially infections with coxsackievirus B (CV-B) (*Human enterovirus* B), are the most suspected environmental factors involved in the development of chronic diseases such as T1D [4,5,6,16]; however, the precise etiology and the mechanisms that trigger virus-induced autoimmunity against islet antigens are not fully understood.

Indeed, after initial replication in the gastrointestinal mucosa, CV-B spreads into the bloodstream through the lymphatic system and reach target organs [17]. The frequent detection of enteroviral components (protein and RNA) in the serum, monocytes, gut mucosa and pancreas as well as anti-CV-B antibodies in saliva of diabetic patients supports the role of persistent infection in the pathogenesis of T1D [18,19,20,21,22,23,24,25,26,27,28,29]. CV-Bs are able to establish a persistent infection in beta cells for up to several years with low levels of viral replication [30,31]. This chronic infection promotes inflammation and innate immunity resulting in insulitis and progressive destruction of insulin-producing cells by preexisting cytotoxic T cells [32]. T1D is believed to be a chronic T cell-mediated autoimmune disease against pancreatic beta cells but other immune cells such as B cells, macrophages, dendritic cells and Natural killer (NK) cells may also be involved in its pathogenesis.

Chronic CV-B4 infection of human pancreatic islets can activate the production of interferon (IFN)-α and IFN-β (by the double-stranded RNA generated during viral replication) and can trigger insulitis with a predominant NK cells infiltration in the early phase of T1D [29,30,33,34]. Viral persistence may be due to a successful evasion from the host immune system leading to viral pathogenesis. Virus-infected cells can escape recognition and destruction by cytotoxic T cells by developing various strategies including the inhibition of the expression and/or function of HLA class I antigens [35]. In contrast, cells with abnormal cell surface expression of HLA class I antigen can nevertheless be recognized and killed by NK cells.

NK cells are innate effector lymphocytes which contribute to the host’s first line of defense against viruses based on their cytolytic activity towards infected cells and their interactions with the innate and adaptive immune system through their capacity to produce a variety of cytokines such as IFN-γ following their activation [36,37,38]. The cytolytic activity of NK cells is modulated by a balance between activating and inhibitory signals transduced via interactions between target cells and NK cell surface receptors [35,39]. The altered numbers, phenotypes and functions of NK cells have been frequently reported in type 1 diabetic patients [40,41,42,43,44,45,46,47]. Moreover, cell-mediated cytotoxicity of NK cells towards various cells infected with CV-B including pancreatic beta cells have been described in animal and human studies which suggest that the defective clearance of pancreatic beta cells infected with CV-B could influence the viral persistence and the susceptibility to virus-induced islet autoimmunity in T1D [31,36,48,49,50].

In this review, the issue of the role of NK cells response to CV-B infection in the pathogenesis of T1D is addressed.

## 2. Biology of Human NK Cells

Human NK cells are granular and large bone marrow-derived lymphocytes, classified as a component of the innate immune system. Even though they act as innate immune system sentinels, they also exhibit characteristics of the adaptive immune system cells such as pathogen-specific cell expansion, generation of long-lasting memory cells and possibility to induce an increased secondary recall response to re-challenge [51,52,53]. NK cells are thymus-independent and do not require preimmunization to exert effector functions [54,55,56,57]. They constitute 5–15% of all circulating lymphocytes in humans [58,59,60] and are phenotypically identified by their expression of CD56 and lack expression of CD3 (CD56^+^CD3^−^ cells) [53,58].

**Figure 1:**
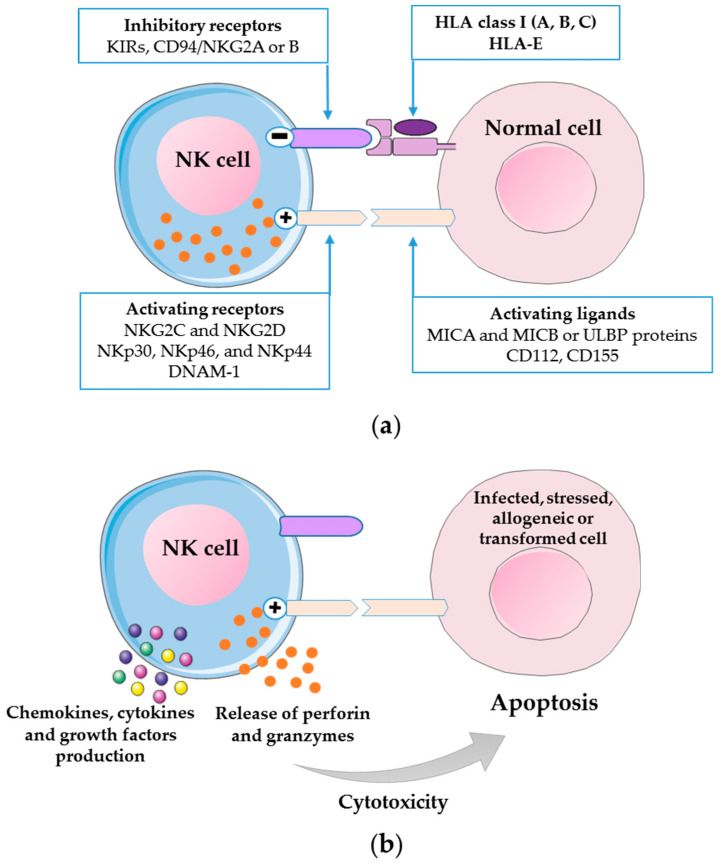
Inhibition and activation of NK cells towards normal and target cells. The cytolytic activity of NK cells is modulated by a balance between activating and inhibitory receptors. (**a**) NK cells towards normal cells. NK cells express inhibitory receptors specific for classical and non-classical Human Leukocyte Antigen (HLA)-I molecules on normal cell. This direct inhibitory interaction induce tolerance towards normal cell by preventing NK cell activation and killing even when NK cell activating receptor binds an activating ligand. (**b**) NK cells towards target cells. Virus-infected, stressed, allogeneic or transformed cells are characterized by downregulation of HLA class I molecules and by an enhanced expression of activating ligands on their surface which promote NK cell activation, leading to cytolysis and killing of these cells by apoptosis through the exocytosis of cytotoxic granules (perforin and granzymes). NK cells also act to promote or suppress functions of innate and adaptive immune cells through their production of chemokines, cytokines and growth factors following their activation by target cells.

NK cells exert recognition and cell-mediated killing against infected, stressed, allogeneic or transformed cells by apoptosis through the release of cytotoxic granules (perforin and granzymes) or through death-inducing receptors pathway via TNF-related apoptosis-inducing ligand (TRAIL) receptor-1 and -2, TNF receptor-1, Fas/APO-1 (CD95) and TNF-receptor-related apoptosis-mediated protein (TRAMP) [39,61,62,63,64]. Their cytolytic activity is modulated by a balance between their germ-line encoded/non-rearranged activating and inhibitory NK cell receptors (NKRs) expressed on cell surface which ensure the capability to either spare normal cells or to kill virus-infected, transformed and/or foreign cells (Figure 1) [35,39,62,65,66,67].

Under homeostatic conditions, NK cells are principally in a resting state due to their inhibitory NKRs which detect the absence of self-molecules on potential target cells (detection of “missing self”) but they can infiltrate tissues or quickly reach the target organs following stimulation by cytokines in pathologic conditions [53,68,69]. Inhibitory NKRs comprise, among others, Killer Immunoglobulin-like receptors (KIRs), which primarily recognize classical Human Leukocyte Antigen (HLA)-I molecules HLA-A, -B and -C; the C-type lectin receptors, which include CD94/NKG2A or -B receptors recognizing the non-classical HLA class I molecule HLA-E; Ig-like transcripts (ILTs); and the leukocyte Ig-like receptors (LIRs) (Figure 1) [66,70,71,72].

NK cells engage cell-mediated killing of virus-infected, tumor transformed or allogeneic non-self-cells that have downregulated surface expression of HLA class I molecules in an effort to avoid recognition by CD8+ cytotoxic T lymphocytes, via the engagement of their activating NKRs including C-type lectin receptors (NKG2C and NKG2D specific for the stress-inducible MICA and MICB or ULBP proteins), Natural Cytotoxicity Receptors (NCRs) (NKp30, NKp46 and NKp44) and DNAM-1 (Figure 1) [65,73,74,75,76].

In addition to cell-mediated cytotoxicity, NK cells also act to promote or suppress functions of T cells, B cells and dendritic cells after interaction with susceptible target cells by producing chemokines (MCP-1, MIP1-α, MIP1-β, RANTES, lymphotactin and IL-8) and both proinflammatory and immunoregulatory cytokines such as IFN-γ, IL-5, IL-10, IL-13, IL-22 and tumor necrosis factor–α (TNF-α) and growth factors such as GM-CSF (granulocyte macrophage colony-stimulating factor), G-CSF (granulocyte colony-stimulating factor) (Figure 1) [64,77,78,79,80,81,82,83,84]. In turns, NK cells can be activated by monocyte-derived cytokines such as IL-1, IL-10, IL-12, IL-15 and IL-18 produced during the innate immune response [58].

Based on their level of CD56 cell-surface expression and their functional features, two major subsets of human NK cells can be identified: CD56^dim^ and CD56^bright^ cells [51,85]. CD56^dim^ NK cells constitute about 90% of the NK cells in peripheral blood and express low level expression of CD56 and high levels of CD16 (FcγRIII) (CD56^dim^CD16^bright^), whereas only 10% of NK cells in blood are CD56^bright^CD16^dim/−^; however, they more easily leave blood vessels and are more abundant in secondary lymphoid tissues, in particular in lymph nodes, tonsils or in chronically inflamed tissues and placenta [86,87,88,89,90,91,92]. Otherwise, some studies showed that human NK differentiation progresses from a CD56^bright^ to a mature CD56^dim^ phenotype following stimulation with IL-2 and IL-15 [58,67,93,94,95,96,97,98]. CD56^dim^ NK cells express a high-level of KIRs and low-level expression of CD94/NKG2A inhibitory receptors. They are highly involved in natural cytotoxicity and antibody-dependent cellular cytotoxicity (ADCC) and produce little amounts of NK-derived cytokines such as IFN-γ. By contrast, CD56^bright^ NK cells are characterized by low expression of KIRs and high-level expression of CD94/NKG2A inhibitory receptors and produce high levels of cytokines with only a weak role in natural cytotoxicity and ADCC [47,58,86,99]. CD56^bright^ NK cells appear to have a high proliferative response to low doses of IL-2 in vitro and in vivo, due to their expression of the high-affinity heterodimeric interleukin-2 receptor (IL-2Rβγ), contrary to CD56^dim^ NK cells [58,100,101,102,103].

## 3. Role of NK Cells in Enteroviral Pathogenesis of T1D

Type 1 diabetes is considered to be a Th1 cell-mediated autoimmune destruction of pancreatic beta cells. Destructive autoimmunity is initiated in steps over years before the clinical onset of diabetes [34,104]. After a release of self-antigens from the target organ/tissue, a priming phase in secondary lymphoid organs is initiated, followed by immune cell infiltration or homing to the target organ/tissue and finally progressive cells or tissue destruction [105,106,107]. Increasing evidence coming from studies of autoimmune diseases suggests that NK cells are probably implicated in all these phases either in the promotion or in the protection against loss of self-tolerance between β-cell antigens and auto-reactive T-cells [45,54,55,59,108,109,110].

The involvement of NK cells in the pathogenesis of T1D is supported by extensive studies performed both in animal models of islet autoimmunity and in human diabetic patients. Indeed, defects in intraepithelial NK cells number and function, including decreased NK cell cytotoxicity precede onset of autoimmune diabetes in diabetes-prone BB rats as well as in LEW and BN rats [111]. NK cell infiltration was increased in the aggressive insulitis of BDC2.5/NOD mice after the blockade of costimulatory molecule such as CTLA-4 (cytotoxic T lymphocyte antigen 4) and depletion of NK cells in this model prevents diabetes development, suggesting their involvement in beta-cell destruction at early-onset diabetes [112]. A study performed by Brauner et al. in NOD mice showed that pancreatic NK cells infiltrated progressively the islets of Langerhans before T cells in younger mice and were hyporesponsive later compared with spleen NK cells, which could reflect exhaustion or regulation after initiation of the inflammatory process in the pancreas [113]. In this line, Ogasawara et al. showed an impairment of activating receptor NKG2D in NK cells of NOD mice, which resulted in down-modulation caused by exposure to NKG2D ligands in the pancreatic islet cells [114]. Flodström et al. observed in transgenic NOD mice expressing the suppressor of cytokine signaling-1 (SOCS-1) (a negative regulator of IFN signaling in insulin secreted cells) that coxsackievirus-B4 infection can induce an acute form of autoimmune diabetes including early and severe hyperglycemia and insulitis with loss of insulin beta cells [115]. In this animal model of coxsackievirus B4-induced diabetes, depletion of NK cells, but not that of CD8+ T cells, prevented beta cell destruction and reduced islet inflammation which demonstrated that CV-B4 infection increased susceptibility to NK cell-mediated killing of beta cells in vivo [115].

A protective role of NK cells in T1D development has also been suggested in preventive or therapeutic approaches. NK cells mediate the protective effects from autoimmune diabetes in NOD mice under complete Freund’s adjuvant treatment through the secretion of IFN-γ and downregulation of beta-cell-specific auto-reactive cytotoxic T lymphocytes [116,117,118]. Moreover, NK cells exhibit an NK3-like phenotype in NOD mice treated with polyinosinic-polycytidylic acid (poly(I:C)), which is involved in prevention of diabetes development through the promotion of Th2 bias of immune responses [119].

Studies on involvement of NK cells in human type 1 diabetes report conflicting results and present limitation regarding the use of pancreatic material from long-established diabetes patients and use of peripheral blood samples that might not reflect the autoimmune process in pancreatic islets during the pre-diabetic phase. A reduction of NK cells frequency in peripheral blood of recent-onset type 1 diabetic patients [41,42,43,46], but not in long-established diabetes patients [46], as compared to healthy individuals, has been reported. On the other hand, some studies have also observed a lower proportion of NK cells in peripheral blood of long-standing type 1 diabetic patients compared to control subjects [31,40,41,44]. A study of Akesson et al. on latent autoimmune diabetes in adults (LADA) suggested that a reduction of NK cell proportion in peripheral blood of these patients could be related to increasing of NK cell number in the pancreas and draining lymph nodes [120]. In regards to the role of NK cell effector functions in T1D, studies have reported conflicting results despite the use of the same myeloid cell line K562 as a NK-sensitive target to evaluate NK cells cytolytic activity of T1D patients. Indeed, some investigations have described an impaired cytolytic activity of these cells in patients with T1D irrespective of the duration of the disease and particularly in long-standing diabetics [43], while others have reported a decreased NK cell cytotoxic activity only in newly diagnosed T1D patients [42]. Oppositely, another study has found a significant increase of NK cells activity in recently diagnosed diabetic patients as compared to long-established diabetic patients and healthy individuals [41].

NK cells of long-established T1D patients respond poorly to IL-2 and IL-15 stimulation and exhibited decreased IFN-γ secretion and NKG2D-dependent cytotoxicity [44]. Further, reduced levels of NKG2D were detected in NK cells of diabetic patients independently of disease duration [46], similarly to the observation in NK cells of the NOD mice [114]. In addition, long-standing T1D patients displayed a reduced NK cells activity manifested by lower expression of surface activating receptors NKp46 and NKp30, a reduced IFN-γ expression and perforin mRNA levels as compared to controls [46]. These observations suggest that NK activity abnormalities especially in long-standing T1D patients, seem to be a consequence of metabolic disorders after the process leading to beta-cell destruction [46], a consequence of immunomodulatory/immunosuppressing effects of insulin treatment [121,122,123,124,125,126] or a consequence of exhaustion of NK cells, which can be observed in the course of chronic infection [127].

The viral pathogenesis of T1D results from interplay between the enteroviruses, innate and adaptive immune response and genetic predisposition [4,6]. Coxsackieviruses B might initiate pathogenic processes through multiple infections or persistent viral infection in the pancreas, blood or gut mucosa cells, which could induce a prolonged inflammatory response, beta cell antigen presentation and destruction of beta cells by preexisting antigen-specific CD8 cytotoxic T cells [16,24,128].

Data on association between enteroviral infection, NK cells, insulitis, autoimmunity and destruction of insulin-producing pancreatic islet beta cells in human are scarce but relevant.

Dotta et al. reported that pancreatic β cells of recent-onset T1D multiorgan donors were infected with CV-B4 [29]. Loss of beta cells function and nondestructive insulitis were found in CV-B4-infected islets with predominant presence of NK cells and, to a lesser extent, of T and B lymphocytes [29]. This study hypothesized that the lack of beta cell destruction could be explained by the absence of autoreactive T cells among the infiltrating leukocytes due to immunoregulatory cytokine IL-10 detected in diabetic infected-islets [29]. This hypothesis is supported by studies demonstrating that NK cells can exert regulatory function including reduction of inflammatory process by producing a significant amount of IL-10 in response to viral infection early [129,130]. In this line, Hofmann et al. showed that CV-B3 can escape from host antiviral defense by suppressing proinflammatory cytokines and inducing IL-10 production, leading to defective viral clearance, persistent infection and chronic myocardiopathy [131].

A recent study by our team described the role of human NK cells in CV-B4 persistence in pancreatic β cells and in the pathogenesis of T1D [31]. We found that CV-B4 can establish a persistent infection in human pancreatic beta cells up to several months with low levels of viral replication [31]. As compared to healthy subjects, an impaired cytolytic activity of IL-2-activated NK cells from patients with T1D towards infected beta cells was observed which suggests that a defective viral clearance by NK cells of patients with T1D may play a role in the persistence of enteroviruses reported in these patients and thus in the viral pathogenesis of T1D [31].

In these clinical studies, all T1D patients were compared to age and sex-matched control subjects and no data was available regarding correlation or relationship between subject age or sex and the profile of NK cells in enteroviral pathogenesis of T1D. Table 1 summarizes the age, sex and conflicting results regarding NK cells number and function of T1D patients in the clinical studies.

## 4. Possible Mechanisms of NK Cells Involvement in CV-B-Induced T1D

Although NK cells are believed to be involved in the pathogenesis of T1D, the mechanisms underlying their role in virus-induced auto-immunity against islet antigens are not fully clarified. The autoimmunity towards pancreatic islets may be initiated both by the release of β-cell antigens due to NK cell cytolytic activity towards infected beta cells or by the production of early source of cytokines affecting adaptive immune response [45,47,54,55,115].

NK cells express inhibitory receptors which recognizes classical HLA class I molecules on target cells [47]. Thus, altered or downregulated surface expression of HLA class I molecules on target cells can induce spontaneous NK cell-mediated killing [35,47]. It was described that many of the picornavirus non-structural proteins as well as their precursors can trigger the rearrangement of intracellular membranes into vesicles which provide platform of positive and negative stranded viral RNAs production [17,132], and thus they inhibit protein trafficking from endoplasmic reticulum to Golgi, resulting in an impairment of HLA class I expression [133,134,135]. Some investigations on CV-B3 acute infection of HeLa cells reported that protein 3A of CV-B3 disrupted the Golgi complex to inhibit anterograde transport, while 2B and 2BC proteins inhibited protein traffic through the Golgi complex and upregulated HLA class I molecules endocytosis, which remove these proteins from the cell surface [50,136,137,138]. In this line, another study showed that CV-B4 can also inhibit expression of HLA class I molecules on human pancreatic beta cells after a persistent infection but not during acute infection through an impact on intracellular protein trafficking rather than on transcriptional process [31]. In such condition, pancreatic beta cells persistently infected with CV-B4 were remarkably lysed by NK cells via apoptosis which could lead to the release of beta-cell antigens and then to autoimmunity [31]. Indeed, evidence support that apoptotic beta cells can direct immune response toward autoimmunity in T1D [139]. Apoptotic beta cells are the most important source of autoantigens. Thus, enhanced beta cells apoptosis or defective apoptotic beta cells clearance by phagocytes can contribute to the autoimmune process in T1D through permanent auto-reactive lymphocyte activation [139,140,141,142].

On the other hand, Hühn et al. suggested that the early human NK cell response to CV-B infection was associated to a high production of IFN-γ rather than cytotoxicity [50]. It has been shown that overexpression of IFN-γ can promote autoimmunity in mice [143,144].

In addition to their antiviral activities resulting in reduction of permissiveness of cells to viral infection and replication described during CV-B infection of human pancreatic islet cells [30,115,145,146,147,148,149], IFN-α and IFN-γ can also enhance NK cells cytolytic activity and upregulate HLA class I expression on islet endocrine cells [34,150,151,152,153,154,155,156]. Thus, during enteroviral infection of genetically susceptible individuals, the possible activation of local NK cells by IFN-α in islets can be a primary cause leading to infected beta cells cytolysis, release of beta-cell antigens and hence activation of antigen-specific CD8 cytotoxic T cells, which is consistent with studies reporting that IFN-α expressed in insulin-producing beta cells can induce T1D in transgenic mice and that beta cells are especially sensitive to NK cell-mediated killing in BB/W diabetic and diabetes-prone rats [157,158,159].

NK cells are an important source of IFN-γ during first hours of innate immune response to infection [60,160], and it has been reported that CV-B infections induce pro-inflammatory cytokines secretion including IFN-γ in insulin-producing cells [161]. However, IFN-γ can also contribute to pancreatic beta cell destruction by direct cytotoxicity and upregulation of HLA class I expression on beta cells leading to increase immune recognition and activation of autoreactive T cells [162,163,164,165,166,167]. This hypothesis is supported by reports demonstrating that overexpression of suppressor of cytokine signaling-1 (SOCS-1), repressing IFN-γ signaling in beta cell lines, protects from CD8+ T cell-mediated autoimmune destruction of pancreatic beta cells in virus-induced T1D [166,168].

In contrast, it has been shown that NK cell-derived IFN-γ can protect mice from the development of CV-B4-induced chronic pancreatitis, by suppressing the effector function of CD8+ T cells involved in the immunopathology without alteration of viral replication which suggests the role of NK cells in viral persistence [169].

**Figure 2:**
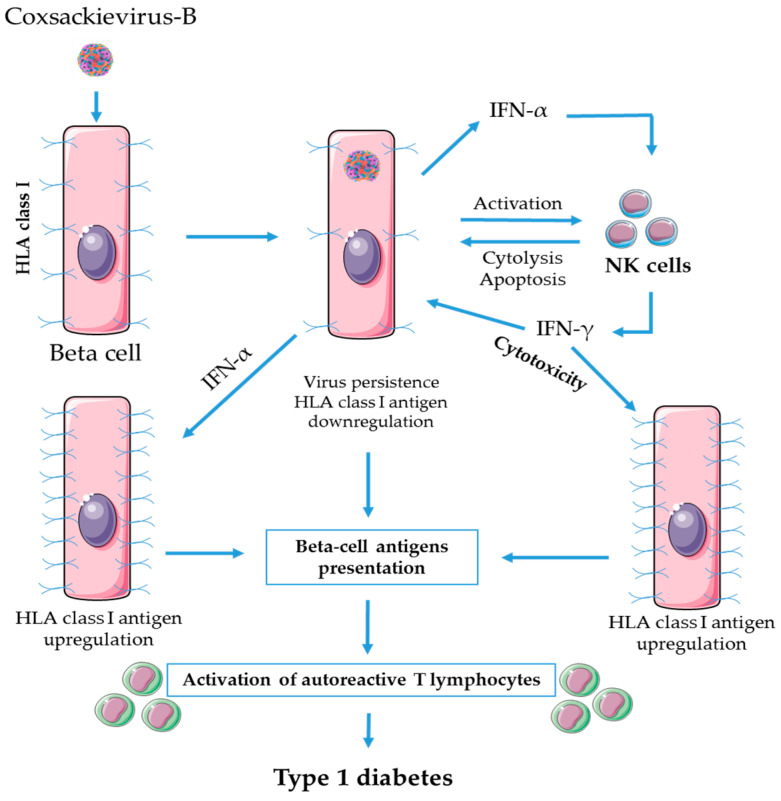
NK cells in the pathogenesis of virus-induced type 1 diabetes during persistent CV-B infection: possible mechanisms. Persistent CV-B infection inhibits the expression of HLA class I molecules on human pancreatic beta cells. NK cells induce apoptosis of virus-infected cells that have altered or downregulated surface expression of HLA class I molecules. Apoptotic beta cells can direct immune response towards autoimmunity through autoreactive T lymphocyte activation. Moreover, IFN-α produced by infected beta cells enhances NK cells cytolytic activity while IFN-γ produced by NK cells damages beta cells (infected and non-infected) by direct cytotoxicity. IFN-α and IFN-γ are able to upregulate HLA class I expression on beta cells leading to increase immune recognition and activation of autoreactive T cells and hence autoimmunity towards beta cells.

Figure 2 shows some aspects of the interplay between CV-B persistent infection, beta cells and NK cells possibly involved in the pathogenesis of T1D.

## 5. Conclusions

Epidemiological and experimental data suggest that an interaction between enteroviruses and innate and adaptive immune system in genetically predisposed individuals can play a role in beta cells alteration and consequently in T1D pathogenesis. NK cells contribute to the host’s first line of defense against viruses but abnormalities in the number and cytolytic activity of these cells as well as their cytokine secretion may affect adaptive immune response and trigger autoimmunity towards pancreatic islets by various nonexclusive mechanisms.

**Figure 3:**
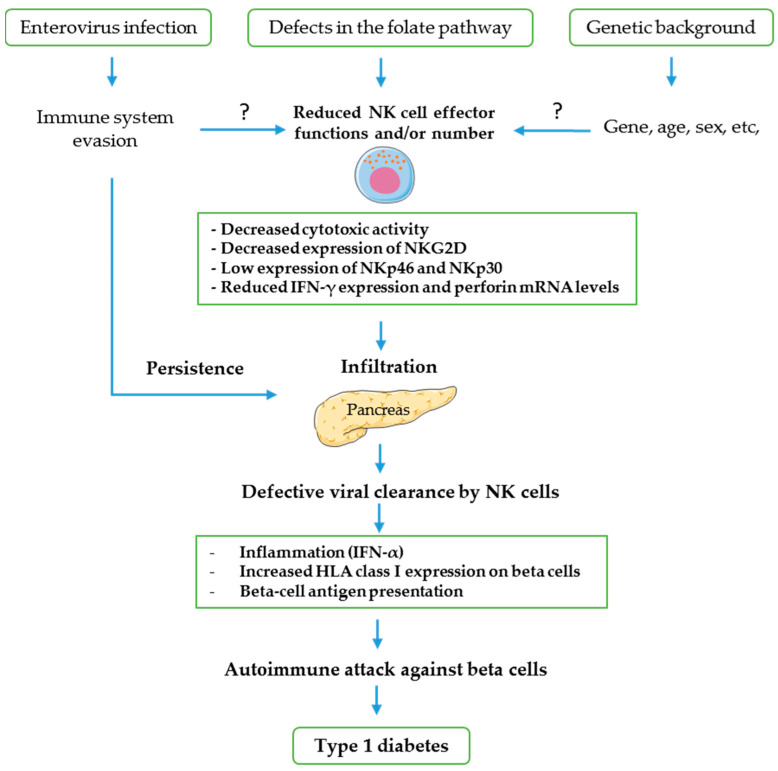
Enteroviruses have demonstrated the capacity to escape host antiviral defense by affecting NK cell effector response through unclear mechanisms. It has also been suggested that defects in folate pathway and a genetic background can play a role in reduction of number and activation of NK cells in T1D patients but the mechanisms are not fully understood. Therefore, a defective viral clearance by NK cells may play a role in the persistent enteroviral infection of pancreas which could induce a prolonged inflammatory response, beta cell antigen presentation and destruction of beta cells by preexisting antigen-specific CD8 cytotoxic T cells leading to T1D.

Nevertheless, some key questions remain to be addressed according to current literature data regarding the causes of the reduced number and activation of NK cells in T1D patients. It cannot be excluded that a genetic background, escape strategies of enteroviruses from host antiviral defense and defects in folate pathway are involved in these NK cells abnormalities in T1D patients (Figure 3) [41,131,170,171].

An improved knowledge of the role of NK cells in host antiviral defense against CV-B infection is required to understand the susceptibility to virus-induced T1D. Furthermore, it could also contribute to the design of new preventive or therapeutic approaches to reduce T1D risk or delay disease onset and progression.

## Figures and Tables

**Table 1:** Summary of findings of clinical studies on NK cells number and function in patients with T1D compared to healthy controls.

Reference	T1D Patients Number (Male/Female)	Age Mean (SD or Range)	NK Cells Frequency in Peripheral Blood of T1D Patients as Compared to Healthy Individuals	NK Cell Effector Functions of T1D Patients as Compared to Healthy Individuals
Nekoua et al. 2020 [31]	7 (2/5)	37.0 years (16.7)	Reduced number of NK cells in long-established T1D patients	Decreased cytotoxic activity in long-established T1D patients. Target cells: CV-B4 persistently infected human 1.1B4 pancreatic beta cells
Hussain et al. 1987 [41]	34 (18/16)	38 years (2–78)	Reduced number of NK cells in recent-onset and long-established T1D patients	Increased cytotoxic activity in recent-onset T1D patients. Target cells: human myeloid K562 cell line
Negishi et al. 1986 [42]	20 (11/9)	12 years (4–35)	Reduced number of NK cells in recent-onset T1D patients	Decreased cytotoxic activity in recent-onset T1D patients. Target cells: human myeloid K562 cell line
Lorini et al. 1994 [43]	25 (14/11)	12.2 years (4.45)	Reduced number of NK cells in recent-onset and long-established T1D patients	Decreased cytotoxic activity in recent-onset and long-established T1D patients. Target cells: human myeloid K562 cell line
Qin et al. 2011 [44]	116 (67/49)	9.3 years (4.5)	Reduced number of NK cells in long-established T1D patients	Decreased IFN-γ secretion and cytotoxic activity and NKG2D-dependent cytotoxicity in long-established T1D patients. Target cell lines: K562, Raji and Daudi
Rodacki et al. 2007 [46]	133 (67/66)	13.6 years (6.55)	Reduced number of NK cells in recent-onset T1D patients	Decreased expression of NKG2D in recent-onset and long-established T1D patients Low expression of NKp46 and NKp30, reduced IFN-γ expression and perforin mRNA levels in long-established T1D patients
